# Punishing Children

**Published:** 1877-06

**Authors:** 


### PUNISHING CHILDREN.

The public sense has been horrified on several occasions of late, by the harrowing details of children, from three to twelve years of age, being so severely punished as to die outright under the infliction, by the hands of their own parents and other protectors. It is scarcely possible that any of our readers need to be warned against such an immeasurable crime, but it is necessary to put parents on their guard in the matter of correcting their children, for no one can tell but that in a moment of crazed phrensy a blow may be struck which may maim for life, or may kill outright on the instant. It is said that the very sight of blood or the taste of a drop induces such an ungovernable thirst for more in a wild beast, as to fill it with ungovernable fury, and cases are given in standard medical works where human beings, after having struck a fellow mortal a deadly blow, have beaten the unresisting body into a jelly after it was known to be dead ; not for the fear of its coming to life again to tell the tale, but from an utterly ungovernable and fiendish impulse, which they could not themselves account for after their minds had become composed.

In multitudes of cases parents have made hasty, and furious and utterly groundless charges against a child, which has so taken it aback that it could make no reply, and this has been taken as a tacit confession of guilt, and the next question proposed in fury is " what did you do it for ?" and the child being thus confused and more alarmed, cannot summon presence of mind and composure enough to make a denial, and as the only alternative bursts into a kind of hysterical crying Many parents are of such a temperament that when a child cannot be induced to utter a word under a scolding, become more enraged, and utter threats which are a disgrace to civilization; we have heard them, ourselves, from affectionate, indulgent and Christian parents, "I'll knock you down with a log of wood." "I'll break every bone in your body." "I'll beat you within an inch of your life," and other similar beastialities of expression from educated, civilized minds, at least they passed for such in the great world.

There is one safe rule always, applicable in the reproof of children; never speak so loud to them that a third person, ten feet away, could hear what was said. Any angry feeling is intensified by a loud utterance. Another good rule is, do not reprove or correct a child in the presence of any third person, or if so, let it be done in a soft, low, affectionate tone. A third precaution, and it is not a minor one either, is, do not reprove on the instant; wait a few hours, if not until next day, or better still in many cases, defer it until the occasion is about to occur when the fault might likely be repeated. Any intelligent and observant housekeeper knows that if a steak is put on the table this morning burned to a crisp, bouncing up from the table, running into the kitchen, and blazing away at the cook is neither ladylike, nor wise, nor [illegible] but next morning just before the steak is about to be cooked, be in the kitchen and ask that it be not overdone as yesterday, with some word of encouragement; whatever servant is not managed in this way had better be dismissed. Now children are as ignorant as servants; the minds of both are weak and may be easily made perverse alike. Be assured, reader, that if you make it an inflexible rule never to scold above a whisper, you will never outrage your child's feelings, nor fracture its skull by a blow dealt in ungovernable fury.

